# Clinical efficacy of immunotherapy combined with chemotherapy in patients with advanced gastric cancer, its effect on nutritional status and Changes of peripheral blood T lymphocyte subsets

**DOI:** 10.12669/pjms.37.7.4347

**Published:** 2021

**Authors:** Wen-wen Li, Jin Jiao, Zhi-yu Wang, Ya-ning Wei, Yuan-fang Zhang

**Affiliations:** 1 Wen-wen Li, Department of Oncology, Affiliated Hospital of Hebei University, Baoding, Hebei 071000, P.R. China; 2 Jin Jiao, Department of Oncology, Affiliated Hospital of Hebei University, Baoding, Hebei 071000, P.R. China; 3 Zhi-yu Wang, Department of Oncology, Affiliated Hospital of Hebei University, Baoding, Hebei 071000, P.R. China; 4 Ya-ning Wei, Department of Oncology, Affiliated Hospital of Hebei University, Baoding, Hebei 071000, P.R. China; 5 Yuan-fang Zhang, Department of Oncology, Affiliated Hospital of Hebei University, Baoding, Hebei 071000, P.R. China

**Keywords:** Immunotherapy, Chemotherapy, Advanced gastric cancer, Nutritional status, T lymphocyte subsets, Cellular immunity

## Abstract

**Objectives:**

To evaluate the clinical efficacy of immunotherapy combined with chemotherapy in patients with advanced gastric cancer and its effect on nutritional status and changes of peripheral blood T lymphocyte subsets.

**Methods:**

Sixty patients with locally advanced gastric cancer who were admitted by Affiliated Hospital of Hebei University from March 2020 to February 2021 were enrolled and randomly divided into two groups, with 30 cases in each group. The control group was treated with FOLFOX4 chemotherapy, while the experimental group was additively treated with cindilizumab on the basis of control group. The incidence of adverse reactions, clinical efficacy, improvement of nutritional and physical status, and changes in the levels of T lymphocyte subgroups in the two groups were compared and analyzed.

**Results:**

The total effective rate was 70% in the experimental group, which was better than 43.3% of the control group (*p*=0.04). The improvement rate of performance status (ECOG) score and nutritional indicators in the experimental group was significantly better than that in the control group (*p*<0.05). Moreover, the indicators of CD3+, CD4+, CD4+/CD8+ in the experimental group were significantly higher than those in the control group after treatment, with statistically significant differences (CD3+, *p*=0.01; CD4 +, *p*= 0.02; CD4+/CD8+, *p*=0.01).

**Conclusion:**

Immunotherapy combined with chemotherapy has a significant effect on locally advanced gastric cancer patients, with significant improvement in physical strength and nutritional status, significant improvement in T lymphocyte function, and no obvious adverse reactions. It is worth promoting in clinical application.

## INTRODUCTION

Gastric cancer, as one of the most common malignant tumors of the digestive tract, features an extremely low overall survival rate.^1^ Surgery is currently considered to be the only radical treatment for gastric cancer. With the progress of surgical techniques and the implementation of traditional radiotherapy, chemotherapy and neoadjuvant therapy, the 5-year survival rate of early gastric cancer can reach > 95%.^2^ However, most patients have no specific symptoms in the early stage, but most of them are locally advanced at the time of treatment, thus missing the optimal surgical window. In view of this, palliative gastrectomy, chemoradiotherapy^3^, molecular targeted therapy and immunotherapy^4^ are the main treatment methods for advanced gastric cancer.

It has been shown in recent domestic and foreign studies that for patients with advanced malignant tumors who receive immunotherapy regimen, their inflammatory response can be decreased, the level of tumor markers will be reduced, and the immune function of T lymphocytes can be improved.^5^ In this study, immunotherapy combined with chemotherapy was used to treat locally advanced gastric cancer patients to observe the efficacy of this combined therapy and the effects on the immune function, physical status and nutritional status of the patients.

## METHODS

### Ethical Approval

The study was approved by the Institutional Ethics Committee of Affiliated Hospital of Hebei University (No.2019Q034; dated: March 1, 2021), and written informed consent was obtained from all participants.

### Patient Information:

### Inclusion criteria

Patients with locally advanced (stage III) gastric cancer; Patients whose lesions can be accurately assessed by CT, MRI and other imaging means; Patients with clear pathological results^6^ and complete clinical data; Patients with good physical condition, able to take care of themselves (KPS score 80), and an expected survival of more than 6 months; Patients who can cooperate to complete the study and have favourable treatment compliance; Patients who have no contraindications to the drugs used in this study.

### Exclusion criteria

Patients with poor general condition and unable to take care of themselves (KPS score<70); Patients with malignant tumors in other parts of the body; Patients with cognitive or behavioral abnormalities who are unable to complete the study; Patients who have taken related drugs that affect the study such as other immunosuppressants, hormones in the near future.

Sixty patients with locally advanced gastric cancer who were admitted by Affiliated Hospital of Hebei University from March 2020 to February 2021 were selected and randomly divided into two groups: the experimental group and the control group, with 30 cases in each group. Among the 60 patients, 17 males and 13 females were grouped into the experimental group, aged 55-70 years old, with an average of 62.34±7.81 years old. Sixteen males and 14 females were grouped into the control group, aged 53-73 years old, with an average of 63.85±7.36 years old. There was no significant difference in general data between the two groups, which were comparable (Table-I).

**Table I:** Comparative analysis of general data between experimental group and control group(X¯±S)n=30.

*Indicators*	*Experimental group*	*Control group*	*t/χ^2^*	*p*
Age	62.34±7.81	63.85±7.36	0.77	0.4331
Male (%)	17(56.7%)	16(53.3%)	0.07	0.80
***Pathological type***				
Papillary adenocarcinoma (%)	13(43.3%)	15(50%)	0.07	0.80
Tubular adenocarcinoma (%)	9(30%%)	10(33.3%)	0.06	0.78
Others (%)	8(26.7%)	5(16.7%)	0.88	0.34
***Tumor location***				
Cardia (%)	13(43.3%)	13(43.3%)	0.00	1.00
Antrum (%)	6(20%)	8(26.7%)	0.37	0.54
Gastric body (%)	6(20%)	5(16.7%)	0.11	0.74
Whole stomach (%)	5(16.7%)	4(13.3%)	0.13	0.72

P>0.05.

### Treatment methods

The control group was given FOLFOX4 chemotherapy regimen^7^: intravenous infusion of 100 mg/m2 oxaliplatin for 2-3 hour on the 1st day; intravenous infusion of 200 mg/m2 calcium folinate for two hour on the 1st day; intravenous infusion of 600 mg/m2 5-fluorouracil on the 1st and 2nd days. A total of 4 cycles of treatment were carried out with 21 days as a cycle.

The experimental group was treated with sindilimab on the basis of the chemotherapy regimen of the control group, i.e., intravenous infusion of 200mg sindilimab once every three weeks.^8^

### Observation indicators

1) Efficacy evaluation: All patients were evaluated for efficacy after every two treatment cycles. Tumors were evaluated according to the Response Evaluation Criteria in Solid Tumors 1.0 (RECIST1.0)^9^: Complete remission (CR): disappearance of all lesion; Partial remission (PR): at least a 30% decrease in the sum of the measured diameters of target lesions from the baseline; Stable disease (SD): a 25%-50% decrease in the longest diameter of lesions; Progression disease (PD): at least a 20% increase in the sum of the long diameters of all target lesions, and at least 5mm increase in the absolute value of the sum of long diameters (or the appearance of one or more new lesions). Total effective rate = number of (CR+PR) cases/total number of cases × 100%.

### Evaluation of adverse drug reactions

Adverse drug reactions after one treatment cycle in both groups were recorded, including red blood cell (RBC) reduction, gastrointestinal reaction, white blood cell (WBC) reduction, liver function injury and other adverse reactions. 3) Improvement of nutritional status and performance status: The ECOG score^10^ was used to assess the physical condition: improvement (≥ 1 point reduction), stability (score unchanged), deterioration (≥ 1 point increase). The changes of nutritional indicators such as hemoglobin, albumin and serum ferritin before and six months after treatment were compared and analyzed. 4) Immune status analysis: early morning fasting blood was drawn before and after treatment to detect the levels of CD3+, CD4+, CD8+, CD4+/CD8+ T lymphocyte subsets.

### Statistical analysis

All the data were statistically analyzed by SPSS 20.0 software, and the measurement data were expressed as (X±s). Two independent sample t-test was used for inter-group data analysis, paired t test was used for intra-group data analysis, and χ^2^ was adopted for rate comparison. *p*<0.05 indicates a statistically significant difference.

## RESULTS

The comparative analysis of the treatment effect of the two groups is shown in Table-II, suggesting that the total effective rate of the experimental group was 70% after treatment, which was significantly better than the 43.3% of the control group, with a statistically significant difference (*p*=0.04).

**Table II:** Comparative analysis of treatment effect between the two groups(X¯±S)n=30.

*Group*	*CR*	*PR*	*SD*	*PD*	*Total effective rate*
Experimental group	6	15	6	3	21(70%)
Control group	5	8	12	5	13(43.3%)
χ^2^					4.34
p					0.04

p <0.05.

The incidence of adverse reactions in the experimental group was 50%, and that in the control group was 36.7%. There was no statistical significance (*p*=0.30). (Table-III).

**Table III:** Comparative analysis of adverse drug reactions between the two groups after treatment(X¯±S)n=30.

*Group*	*RBC reduction*	*Gastrointestinal reaction*	*WBC reduction*	*Liver function injury*	*Incidence*
Experimental group	3	3	5	4	15(50%)
Control group	2	4	3	2	11(36.7%)
χ^2^					0.08
p					0.30

p <0.05.

The improvement rate of ECOG in the experimental group after treatment was significantly higher than that in the control group (*p*=0.03) (Table-III). The nutritional indicators of the two groups after treatment, such as hemoglobin, albumin, serum iron, and ferritin, were increased compared with those before treatment. The improvement degree of the experimental group was more obvious than that of the control group. (Table IV - V).

**Table IV:** Comparative analysis of ECOG before and after treatment between the two groups(X¯±S)n=30.

*Group*	Improvement*	*Stable*	*Deterioration*
Experimental group	17	9	4
Control group	9	13	8
χ^2^	4.34	1.14	0.67
p	0.03	0.28	0.20

* p<0.05

**Table V:** Comparative analysis of the improvement of serum nutritional indicators of the two groups before and after treatment(X¯±S)n=30.

*Group*	*Hemoglobin (g/L)* *	*Albumin (g/L)* *	*Serum iron (mmol/L* *	*Ferritin (ug/L)* *
Experimental group	6.13±2.36	4.57±1.43	7.01±2.20	4.72±2.35
Control group	2.74±1.07	3.72±1.09	5.37±2.13	3.28±2.51
t	7.16	2.58	2.93	2.29
p	0.00	0.01	0.04	0.03

* p <0.05.

The indicators of CD3+, CD4+, CD4+/CD8+ in the experimental group were significantly higher than those in the control group after treatment, with statistically significant differences (CD3+, *p*=0.01; CD4+, *p*=0.02; CD4+/CD8+, *p*=0.01), while CD8+ did not change significantly (*p*=0.79) (Table-VI)

**Table VI:** Comparative analysis of T lymphocyte subsets between the two groups before treatment(X¯±S)n=30.

*ndicators*		*Experimental groupΔ*	*Control groupΔ*	*t*	*p*
CD3+(%)	Before treatment*	43.36±7.58	42.71±7.42	0.34	0.71
	After treatmentΔ	49.47±6.85	46.16±6.03	2.58	0.01
	t	3.27	2.72		
	p	0.00	0.01		
CD4+(%)	Before treatment*	26.37±4.90	26.52±5.47	0.11	0.90
	After treatmentΔ	37.08±5.33	33.71±5.58	2.39	0.02
	t	8.10	5.03		
	p	0.00	0.00		
CD8+(%)	Before treatment*	21.37±3.21	21.57±3.61	0.22	0.82
	After treatment*	21.85±3.36	22.07±3.04	0.26	0.79
	t	0.56	0.86		
	p	0.32	0.23		
CD4+/CD8+	Before treatment*	1.27±0.31	1.25±0.53	1.78	0.85
	After treatmentΔ	1.83±0.55	1.57±0.21	2.58	0.01
	t	2.42	3.07		
	p	0.02	0.00		

* p >0.05, Δ*p*<0.05.

## DISCUSSION

Gastric cancer (GC) is a fatal malignant tumor of the digestive tract, which is difficult to diagnose early.^11^ Surgical resection is given priority in the treatment of early GC. However, with an in-depth understanding of the biology of GC, unresectable advanced GC can be treated with a variety of different therapies, including chemotherapy, neoadjuvant chemotherapy, immunotherapy, and targeted therapy, and a significant increase can be seen in the survival advantage of unresectable advanced GC.^12^

Chemotherapy, among multimodal treatment strategies for GC, plays a unique role in conferring survival benefits.^13^ It has been considered in some studies that an effective chemotherapy regimen can not only effectively kill tumor cells, but also has a significant role in regulating the expression of oncogenes, thereby improving the long-term prognosis of patients.^14^ Gastrointestinal reaction, malnutrition and immune dysfunction are common side effects of chemotherapy.^15^ Chemotherapy drugs have a certain killing effect on T lymphocyte subsets while inhibiting tumor cells.

Certain survival benefits may be brought to patients with advanced GC by the combination of chemotherapy and immunotherapy.^16^ It was considered by Erdogdu Et Al.^17^ that the poor prognosis of advanced GC has a bearing on the increased expression of PDL-1 in cancer cells. PD-1 is mainly expressed on the surface of immune cells, while PDL-1, by contrast, is mainly expressed on the surface of tumor cells. The combination of PD-1 and PDL-1 leads to immune escape of tumor cells by activating signaling pathways in immune cells. It was shown in the study of He et al.^18^ that the killing effect of TIL could be increased by blocking PD-1/PD-L1, thereby increasing the sensitivity of tumors to chemotherapy. Even in advanced GC, CD8+ T cells react in an antigen-specific manner, suggesting that the levels of memory T cells, NK cells and NKT cells in GC tissue have a close bearing on a favourable prognosis.

The function of various immune cells is considered to be extremely important in the treatment of GC. Recent clinical trial data on the efficacy of immunotherapy show that anti-PD-1/PD-L1 inhibitors appear to be beneficial for subgroups of patients with advanced gastric or esophageal cancer who have undergone a variety of systemic chemotherapy^19^. It was also considered in the study of Coutzac et al.^20^ that blocking the PD-1/PD-L1 axis might have anti-tumor effect. In our study, chemotherapy combined with immunotherapy was confirmed to have a total effective rate of 70% for locally advanced GC, which was significantly better than that of the chemotherapy-alone group (*p*=0.04), while the incidence of adverse reactions was not significantly increased compared with that of the chemotherapy-alone group (*p*=0.30). Moreover, the indicators of CD3+, CD4+, CD4+/CD8+ in the experimental group were significantly higher than those in the control group after treatment, with statistically significant differences (*p*<0.05), suggesting that the cellular immune status of patients was significantly improved after chemotherapy combined with immunotherapy.

Balancing the immune status of patients can improve the nutritional status and performance status of patients with gastrointestinal cancer^21^, which is extremely beneficial to the rehabilitation of patients. It was also confirmed in this study that the performance status of the experimental group after treatment was significantly improved than that of the control group(*p*=0.03), and the hemoglobin, albumin, serum iron and ferritin levels in the experimental group improved more obviously than those in the control group, with statistically significant differences (*p*<0.05).

### Limitations of the study

Nevertheless, deficiencies can still be seen in this study: less sample size, short follow-up time, in addition, for the sake of studying the stability of the indicators, T lymphocyte subsets were included in the study, but the blood immunoglobulin content of patients was not analyzed to further confirm the effect of such a regimen on humoral immunity in patients. In view of this, proactive countermeasures are being taken to accumulate cases and relevant experience, and to further increase the study content of humoral immunity, so as to conduct a more comprehensive assessment of the benefits of the treatment regimen for patients.

## CONCLUSION

Immunotherapy combined with chemotherapy has a significant effect on locally advanced gastric cancer patients, with significant improvement in physical strength and nutritional status, significant improvement in T lymphocyte function, and no obvious adverse reactions. It is worth popularizing in clinical application.

### Authors’ Contributions:

**WWL & JJ:** Designed this study and prepared this manuscript,and are responsible and accountable for the accuracy or integrity of the work.

**ZYW & YNW:** collected and analyzed clinical data; Yuan-fang Zhang significantly revised this manuscript.
